# Bilateral Blindness Due to Radiation-Induced Optic Neuropathy in a Patient with Nasal Mucosal Melanoma

**DOI:** 10.22336/rjo.2026.47

**Published:** 2026

**Authors:** Mauricio Agustin Benetti, Luciana León-Cejas, Luis Ariel Miquelini, Dolores Ribero-Ayerza, Francisco Caiza-Zambrano, Ricardo Reisin

**Affiliations:** 1 Neurology Department, Hospital Británico de Buenos Aires, Argentina; 2 Neuroradiology Department, Hospital Británico de Buenos Aires, Argentina; 3 Neuro-ophthalmology Department, Hospital Británico de Buenos Aires, Argentina

**Keywords:** optic neuropathy, radiation-induced, melanoma, mucosal, radiotherapy, immune checkpoint inhibitors, blindness, ACEIs = Angiotensin-converting enzyme inhibitors, CSF = Cerebrospinal fluid, GCL = Ganglion cell layer, ICIs = Immune checkpoint inhibitors, IVIG = Intravenous immunoglobulin, LE = Left eye, MRI = Magnetic resonance imaging, OCT = Optical coherence tomography, PD-1 = Programmed cell death protein 1, RE = Right eye, RION = Radiation-induced optic neuropathy, RNFL = Retinal nerve fiber layer, RT = Radiotherapy, VEGF = Vascular endothelial growth factor

## Abstract

Radiation-induced optic neuropathy is an uncommon but devastating complication of radiotherapy for head and neck tumors, characterized by subacute, painless, irreversible visual loss. Nasal mucosal melanoma, although rare, carries a high risk of visual pathway toxicity due to its proximity to anterior optic structures.

We report the case of a 73-year-old woman with right nasal mucosal melanoma treated with adjuvant radiotherapy (66 Gy) and anti-PD1 immunotherapy, who developed progressive bilateral vision loss. Neuro-ophthalmological examination revealed severe visual acuity reduction, bilateral mydriasis, and optic disc pallor. Optical coherence tomography showed retinal nerve fiber layer thinning. Brain MRI demonstrated contrast enhancement of the optic chiasm and prechiasmatic optic nerves.

Despite intensive treatment, the patient progressed to bilateral blindness.

This case underscores the importance of early recognition of radiation-induced optic neuropathy, particularly in high-risk anatomical sites and in patients receiving radiotherapy combined with immunotherapy.

## Introduction

Radiation-induced optic neuropathy (RION) is an uncommon but potentially devastating complication of radiotherapy (RT) for intracranial or head and neck tumors. It typically presents with subacute, painless, and irreversible vision loss, with a latency of 3 months to 7 years after RT, averaging around 18 months [1]. Although the overall incidence is low, it may reach up to 9% in nasal cavity and nasopharyngeal tumors irradiated with doses exceeding 50 Gy [2].

Nasal mucosal melanoma is a rare and aggressive subtype, accounting for approximately 1.3% of all melanomas [3]. Due to its proximity to the anterior visual pathways, adjuvant RT in this location carries a significant risk of severe visual toxicity. In this context, the increasing use of immune checkpoint inhibitors (ICIs) in head and neck malignancies poses an additional diagnostic challenge in patients presenting with acute vision loss, as immune-mediated optic neuropathy may mimic or coexist with RION [4].

We describe the case of a patient with nasal mucosal melanoma treated with RT and anti-PD1 immunotherapy, who developed progressive bilateral RION culminating in irreversible blindness.

## Case report

A 73-year-old woman presented with progressive visual decline, initially affecting the left eye (LE) and rapidly progressing to painless amaurosis, followed by sequential involvement of the right eye (RE). Her history included tobacco use and a recently diagnosed right nasal mucosal melanoma confirmed by biopsy. She was receiving combined immunotherapy with nivolumab and ipilimumab. Ten months before symptom onset, she had completed adjuvant RT (66 Gy in 33 fractions of 2 Gy) using 6 MV photons via linear accelerator.

**Fig. 1:**
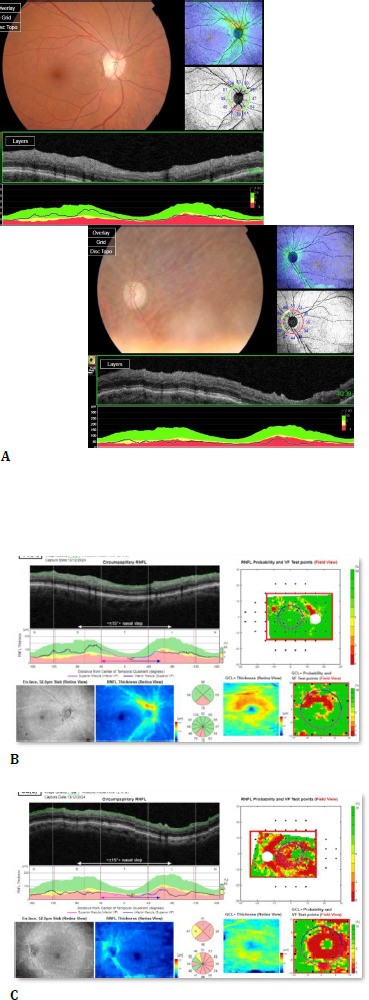
A. Optical coherence tomography (OCT) of the retinal nerve fiber layer (RNFL) of the right eye (RE) shows thinning, predominantly in the inferior quadrant. The left eye (LE) shows persistent diffuse thinning (RE left, LE right); B. OCT of the RE: Sectoral thinning of the RNFL with involvement of the ganglion cell layer (GCL) in the inferior quadrant; C. OCT of the LE: Severe GCL atrophy, consistent with near-complete loss of the layer

Neuro-ophthalmological examination revealed a best-corrected visual acuity of 0.2-0.3 in the RE and no light perception in the LE. The pupillary light reflex was present in the RE and absent in the LE. Fundus examination revealed mild optic disc pallor in the RE and diffuse pallor in the LE. Optical coherence tomography (OCT) demonstrated thinning of the retinal nerve fiber layer (RNFL) in the RE, predominantly in the inferior quadrant. At the same time, the LE showed diffuse thinning consistent with global involvement (Fig. 1).

**Fig. 2:**
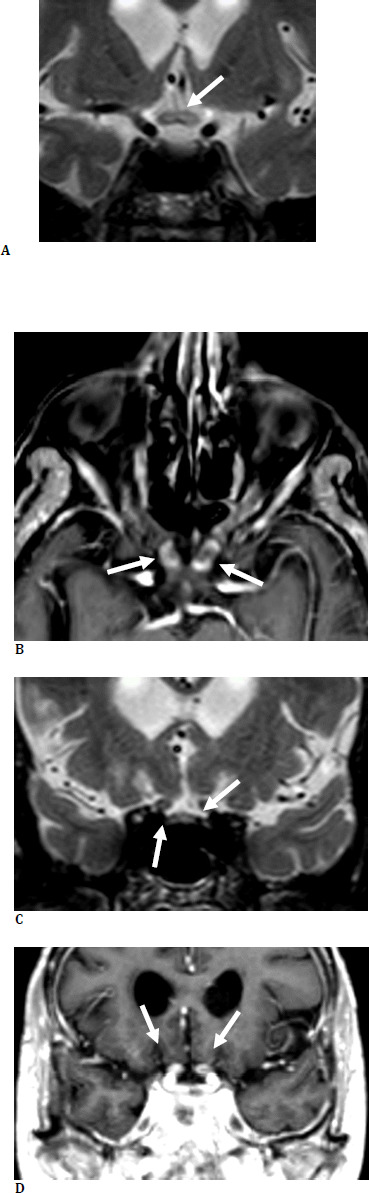
Brain MRI: Axial and coronal sequences showing thickening and STIR hyperintensity of the optic chiasm (A) and prechiasmatic segments of both optic nerves (C) (arrows), associated with contrast enhancement T1-weighted images (B, D) (arrows)

Brain and orbit MRI demonstrated STIR hyperintensity and contrast enhancement of the optic chiasm and prechiasmatic segments of both optic nerves, more pronounced in the left optic nerve, including its intraorbital portion (Fig. 2).

No tumor recurrence was identified.

Visual evoked potentials showed bilateral conduction abnormalities, greater in the left optic nerve.

A lumbar puncture yielded clear and colorless cerebrospinal fluid (CSF) with an opening pressure of 18 cmH_2_O, white cell counts of 5 × 10^6^/L (95% lymphocytes), protein 0.37 g/L, glucose 4.7 mmol/L, and negative flow cytometry and oligoclonal bands. An immunological panel was also negative, with no evidence of specific autoantibodies (anti-CV2/CRMP5 and anti-recoverin) in both serum and CSF.

Given the clinical severity and imaging findings, a presumptive diagnosis of RION was established. Other causes of acute bilateral optic neuropathy were excluded.

The patient received treatment with intravenous methylprednisolone (1 g/day for 5 days), followed by intravenous human immunoglobulin (IVIG, 0.4 g/kg/day for 5 days) and 25 sessions of hyperbaric oxygen therapy at 2.4 atmospheres absolute as part of the escalation treatment strategy.

Despite this therapy, the patient developed amaurosis of the RE within 30 days of the initial symptoms in the LE.

The combination of temporal evolution, exclusion of alternative aetiologies, and imaging features supported the diagnosis of RION. Despite timely and aggressive interventions, the patient progressed to irreversible bilateral blindness.

## Discussion

RION is a severe ophthalmological complication observed in patients previously exposed to cranial irradiation, often resulting in irreversible visual impairment with profound impact on functional status and quality of life [5,6]. The principal risk factor for RION is the total radiation dose. The risk is virtually negligible when total doses are <50 Gy, administered in fractions <2 Gy; however, it may reach up to 20% when doses exceed 60 Gy with fraction sizes ≥2 Gy, owing to a dose-dependent mechanism of endothelial injury that leads to ischaemia of the optic nerve [1]. Additional predisposing factors include comorbidities such as diabetes mellitus, hypertension, and concomitant chemotherapy [1,2]. The pathophysiology remains incompletely understood. A predominantly ischaemic mechanism has been proposed, mediated by the accumulation of free radicals causing vascular endothelial damage, leading to occlusive vasculitis and axonal injury. Additionally, a “bystander effect” has been described, wherein non-irradiated adjacent cells sustain secondary injury mediated by intercellular signaling [2,7]. The concept of Lessell’s triad—hypovascularity, hypocellularity, and hypoxia—remains central to explaining radiation-induced neural injury. This triad reflects the progressive obliteration of the microvasculature, loss of supportive glial and vascular cells, and a persistent hypoxic microenvironment, ultimately impairing axonal metabolism and contributing to delayed, irreversible optic nerve damage [7]. Clinically, RION typically presents as acute, painless, and irreversible vision loss, initially monocular, with potential sequential progression to the contralateral eye. During the acute phase, fundoscopic findings may include optic disc edema, hyperemia, peripapillary subretinal fluid, hard exudates, and peripapillary hemorrhages, ultimately progressing to optic atrophy [1,6]. The diagnosis of RION is primarily clinical and should be supported by a comprehensive neuro-ophthalmological evaluation, including best-corrected visual acuity, pupillary light reflex, fundus examination, and OCT of the RNFL and GCL. OCT is particularly useful in detecting progressive RNFL thinning—especially in the superior quadrant—as well as GCL atrophy and transient retinal thickening due to early ischaemic macular oedema [3,6]. MRI typically demonstrates contrast enhancement on T1-weighted sequences in the optic nerve and/or chiasm, accompanied by T2 hyperintensity and segmental enlargement [7-9]. The differential diagnosis must consider tumor progression or recurrence with optic nerve infiltration, as well as arteritic and non-arteritic ischaemic optic neuropathies. In patients receiving immune checkpoint inhibitors (ICIs), immune-mediated optic neuropathy should also be considered [1-6].

In our patient, the bilateral, subacute, and sequential progression of visual impairment, together with the history of adjuvant RT ten months before symptom onset, strongly supported the diagnosis of RION. Unlike ICI-associated optic neuritis, which can present unilaterally or bilaterally and may partially improve with discontinuation of immunotherapy and treatment with corticosteroids or IVIG [10,11], our patient showed no clinical improvement. Moreover, MRI demonstrated bilateral, symmetrical enhancement of the optic chiasm and prechiasmatic segments, in contrast to the focal intraorbital enhancement pattern typically observed in ICI-associated optic neuritis [10-12]. This diagnostic challenge is further compounded by a rare tumor type (nasal mucosal melanoma), rapid bilateral progression over a short interval, and the coexistence of immunotherapy, making our case particularly unusual and increasingly relevant in the era of combined oncological therapies.

To date, no consistently effective treatment for RION has been established. Therapeutic strategies proposed include hyperbaric oxygen therapy within 48-72 hours of the onset of vision loss and before optic disc pallor; systemic corticosteroids; anticoagulants; angiotensin-converting enzyme inhibitors (ACEIs); and, more recently, bevacizumab, an anti-VEGF monoclonal antibody. While promising results have been reported in selected cases, no consistent benefit has been observed in patients presenting with profound vision loss [2,5,7]. In our patient, the lack of response to corticosteroids, hyperbaric oxygen therapy, and IVIG further reinforces the limited efficacy of currently available treatments, highlighting the need for preventive strategies, none of which have been validated to date. In this context, experimental and observational studies have suggested a potential protective role of early administration of bevacizumab or angiotensin-converting enzyme inhibitors (ACEIs), such as ramipril, following irradiation [3]. The visual prognosis in RION remains poor, particularly when treatment is not initiated within the first 72 hours [6]. Exceptionally, RION has been reported after radiotherapy for nasal melanoma [13]. In a reported case, a patient developed bilateral blindness despite corticosteroids and anticoagulation, consistent with the outcome observed in our case, despite a more intensive therapeutic approach.

## Conclusion

We presented a case of RION that progressed to bilateral blindness in a patient with nasal mucosal melanoma. We analyzed the clinical presentation, diagnostic approach, therapeutic options, and prognosis of this condition. Early recognition remains crucial, as timely intervention may mitigate the progression of visual loss in selected cases. The case highlighted the potential visual complications of radiotherapy in anatomically high-risk tumors, particularly when combined with immunotherapy, and the unusual coexistence of nasal mucosal melanoma, ICI therapy, and subsequent RION.
